# Addressing the impact of forest fires in Quito on youth health

**DOI:** 10.3389/fpubh.2025.1532865

**Published:** 2025-01-21

**Authors:** José Francisco López-Gil

**Affiliations:** One Health Research Group, Universidad de las Américas, Quito, Ecuador

**Keywords:** air quality, wildfires, health risks, children, adolescents, community response

The ongoing forest fires in Quito (Ecuador) have brought to light a pressing environmental and public health crisis (1). As the fires rage on, we are witnessing the detrimental effects on air quality, which is rapidly deteriorating due to prolonged droughts. These fires, much like those experienced in certain countries (e.g., United States, Australia, and Portugal) in recent years, pose a serious threat to vulnerable populations (2), particularly children and adolescents (3).

We know from past studies that exposure to poor air quality, as seen during wildfire seasons, can lead to significant health issues (4), especially respiratory problems in children (5). However, beyond the immediate health risks, these environmental disasters also limit opportunities for physical activity, which is crucial for the healthy development of young people (6, 7). For instance, research on the Australian bushfires, for example, demonstrated a sharp decline in physical activity levels among children only when air quality exceeded critical hazardous levels (6).

In Quito, we must be proactive in ensuring that the physical and mental wellbeing of our youth is protected during these times of crisis. In light of the ongoing fires, I would like to offer some evidence-based recommendations to help mitigate the risks to children's health:

- Monitor air quality closely: families should regularly check local air quality levels, especially when wildfires are present. Tools like mobile applications or websites can provide real-time data (8, 9). For instance, Google Maps and Apple Maps now include Air Quality Index (AQI) indicators in the lower corner of the map when searching for locations. Additionally, dedicated apps like AirVisual, Air Quality Index China Network (AQICN), IQAir, or government platforms (e.g., Ecuador's Ministry of Environment, Water and Ecological Transition) often provide accurate air quality updates. By using these tools, parents can decide when it is safe for their children to be outdoors. On days when air quality is poor, outdoor physical activities should be avoided to prevent inhalation of harmful particles (10).
- Promote safe indoor physical activity: it is essential to maintain children's physical activity levels, even indoors (11). Parents can encourage simple, low-cost activities that require minimal equipment, such as dancing to music, creating obstacle courses with household items (e.g., pillows, chairs, or boxes), or playing traditional games like hopscotch or hide-and-seek indoors. Additionally, free online resources, such as YouTube videos offering kid-friendly exercise routines or yoga sessions, can be a great way to keep children active without financial investment. These activities help maintain their physical health, particularly during extended periods indoors (12).
- Manage sedentary behavior: with reduced opportunities for outdoor play, children often resort to screen-based activities for entertainment (13). It is important to establish daily routines that balance screen time with physical movement (12). For instance, short indoor activities, like stretching or light exercises, can break up long periods of sitting. Additionally, if air quality permits, walking indoors or in well-ventilated areas can be a simple yet effective way to promote physical activity. Walking not only helps reduce sedentary behavior but also serves as a natural stress reliever (14), lowering depression and anxiety levels (15) and increasing psychological well-being (16). Encouraging brief family walks, even in small spaces or hallways, could foster both physical and mental well-being.
- Prioritize sleep: poor air quality (7) and increased screen time (17) could negatively affect sleep, which is vital for both physical and mental health (18). Parents should ensure that children have a consistent bedtime routine, limit screen exposure before bed, and create a quiet, comfortable environment for sleep (19). Maintaining regular sleep schedules, even on weekends, helps regulate children's internal body clocks and promotes restorative sleep (20). Additionally, incorporating relaxing pre-sleep activities such as reading (21) or listening to calming music (22) could further improve sleep quality and prepare children for a restful night.
- Mental health support: the anxiety and stress that accompany environmental disasters can have long-lasting effects on the mental health of young people (23). Parents and educators must be vigilant and provide emotional support (24), perhaps by creating routines that include relaxing activities like meditation or family games that engage children mentally while keeping them calm. However, effectively monitoring and supporting mental health requires knowledge and training. Providing access to resources, such as workshops on recognizing signs of anxiety and stress, or online modules on youth mental health first aid, can empower parents and educators to respond appropriately. Schools and communities could also establish partnerships with mental health professionals to offer guidance and create a supportive environment for both children and their caregivers. Incorporating measures such as regular check-ins or the use of validated screening tools [e.g., Strengths and Difficulties Questionnaire, SDQ (25)] could help identify children who might need additional support and ensure timely intervention.
- Healthy eating: a balanced diet can help strengthen children's immune systems (26), particularly during times when physical activity is reduced (27). Providing access to nutrient-rich foods, such as fruits, vegetables, whole grains, lean proteins, and healthy fats, is essential to ensure children receive the vitamins and minerals needed for growth and resilience against illnesses. Foods rich in antioxidants, like berries and leafy greens, could help combat inflammation caused by poor air quality (28), while sources of omega-3 fatty acids, such as fish or walnuts, support brain health and emotional well-being (29). Parents can encourage healthy eating habits by involving children in meal planning and preparation, making it a family activity that fosters positive attitudes toward nutritious foods (30). Promoting these practices helps reduce the risks associated with decreased physical activity while enhancing mental health and overall well-being.
- Prepare household disaster kits: as part of disaster preparedness, families should consider creating or updating their household disaster kits to include resources that support children's physical and mental health (31). In addition to essentials like water, food, and first aid supplies, kits can include items that keep children engaged and active during crises (32). Examples include coloring books, puzzles, age-appropriate games, small exercise tools like resistance bands, and calming objects such as stress balls or sensory toys. Encouraging children to help pack these items can also foster a sense of involvement and security, further supporting their resilience in challenging situations (33). However, although disaster supply kits are widely recommended at a national level, further studies are necessary to better understand their role in enhancing survival rates and resilience after such events (34).

The situation in Quito serves as a reminder of the broader challenges we face with climate change and its impact on public health (35). Protecting our youth requires swift action, informed strategies, and ongoing community engagement to ensure they emerge from this crisis as healthy and resilient individuals (36).
